# Nicotine Dependence and the Level of Motivation for Ceasing Smoking in the Case of Patients Undergoing Vascular Surgeries Versus the Optimisation of Perioperative Care—Pilot Survey

**DOI:** 10.3390/ijerph191610393

**Published:** 2022-08-20

**Authors:** Renata Piotrkowska, Wioletta Mędrzycka-Dąbrowska, Piotr Jarzynkowski, Robert Ślusarz

**Affiliations:** 1 Department of Surgical Nursing, Medical University of Gdańsk, Dębinki 7, 80-211 Gdańsk, Poland; 2 Clinic of Cardiac and Vascular Surgery, University Clinical Centre, Dębinki 7, 80-952 Gdańsk, Poland; 3 Department of Anaesthesiology Nursing and Intensive Care, Medical University of Gdańsk, Dębinki 7, 80-211 Gdańsk, Poland; 4 Neurological and Neurosurgical Nursing Department, Faculty of Health Science, Collegium Medicum in Bydgoszcz, Nicolaus Copernicus University in Toruń, Łukasiewicza 1, 85-821 Bydgoszcz, Poland

**Keywords:** nicotine, smoking, vascular surgeries, perioperative care

## Abstract

Introduction: Smoking is one of key risk factors of cardiovascular diseases, including abdominal aortic aneurysm (AAA), peripheral arterial disease (PAD), and carotid artery disease (CAD). Despite attempts being made to make the society aware of the consequences of passive and active smoking, as well as worldwide and nationwide epidemiologic research reflecting the scale of the problem, there are still a lot of smokers. Aim: The purpose of the study was to assess the relationship between the degree of addiction and the level of motivation for ceasing smoking in the case of patients before vascular surgery. Furthermore, to point out factors that have a significant impact on the level of nicotine dependence and motivation for ceasing smoking. Methods: The survey included patients qualified for vascular surgeries. The patients were active smokers: 69.3% men and 30.7% women. The survey was conducted in the form of a questionnaire based on standard research tools: the Fagerström Test for Nicotine Dependence (FTND), the Test of Motivation for Ceasing Smoking by Nina Schneider, and the original questionnaire aimed at collecting social and demographic data. Results: Most patients showed a high or moderate degree of nicotine dependence: 46.5% and 40.6%, respectively. An average nicotine dependence ratio based on the Fagerström test was 6.23 ± 2.39. An average motivation for the ceasing smoking ratio was 4.88 ± 2.76. Only 34.7% of the patients had a high motivation for ceasing smoking. Over half of the patients (61.4%) attempted to cease smoking in the past. Conclusions: Most patients undergoing vascular surgeries showed a high or moderate degree of nicotine dependence and low motivation to quit smoking. The greater the addiction to nicotine, the lower the motivation to quit smoking. Social and demographic factors do not affect the degree of nicotine addiction and the motivation to quit smoking. Years of smoking had an impact on the incidence of chronic obstructive pulmonary disease (COPD).

## 1. Introduction

### 1.1. Smoking versus Vascular Diseases

Although the frequency of smoking in many western countries has been decreasing, smoking still remains one of the main worldwide reasons for the development of atherosclerosis and cardiovascular diseases [1]. It has been estimated by the World Health Organization (WHO) that 4.9 million people die per year because of smoking, and the death toll is expected to rise to 8 million by 2030 [2]. In Poland, smoking remains one of major reasons for premature deaths. The results of the nationwide research on attitudes to smoking indicate that in 2019 almost 8 million people regularly smoked tobacco in Poland, i.e., 21% of the population of Poland [3,4]. Nicotine, which is the main ingredient of cigarettes, has both prothrombotic and atherosclerotic effects and increases the risk of atherosclerosis [5], as well as vascular diseases, such as coronary heart disease (CHD), abdominal aortic aneurysm (AAA), peripheral arterial disease (PAD), carotid artery disease (CAD) [5,6,7].

Ceasing smoking brings about benefits to all smokers, whatever their age and the number of cigarettes they smoke. This is a key element of the strategy of primary and secondary prevention of cardiovascular diseases [8,9].

Nicotine dependence is connected with stimulating the systems of neurotransmitters in the central nervous system. Stimulating α4β2 receptors increases the release of dopamine. This results in the feeling of pleasure, better mood, the reduction in fear and anxiety, as well as the stimulation of the organism, and even the improvement of memory [10]. Inhaling nicotine from a tobacco product also contributes to the release of noradrenaline (“activity hormone”). When a cigarette is smoked, the concentration of nicotine or noradrenaline in the blood naturally decreases after some time. Then, the smoker feels a nicotine withdrawal symptom, which is unpleasant for the organism. To improve their condition, patients feel a strong need to smoke another cigarette [11]. Patients of surgery wards usually do not smoke during their hospitalisation due to the non-smoking restrictions, but they come back to the addiction the moment they leave the hospital. Treating nicotine dependence is a challenge to medical professionals and patients.

### 1.2. Optimisation of Perioperative Care

The concept of prerehabilitation was created to improve the quality of the perioperative care. The individual elements are implemented at the very beginning of treatment, and their purpose is to improve the general condition of the organism. In accordance with the definition, the prerehabilitation focuses on:

- Taking care of the proper nutrition of the organism;
- Improving the general function of the organism through physical activity;
- Providing psychological support;
- Eliminating harmful addictions: smoking and abuse of alcohol [12].

Smoking is a recognised perioperative risk factor applicable to 25% of patients of surgery wards [12,13]. Smoking before surgery has a toxic impact on the function of lungs, the heart, and the immunological system, as well as wound treatment, and it generates the risk of infection in the operated place. That is why the smokers are also less capable of meeting surgical requirements [13]. Many studies confirm that ceasing smoking should be an intrinsic element of preparation to any surgery. Physiological benefits appear within several minutes from the last cigarette [13,14]. To reduce postoperative complications, the patient must cease smoking 4–6 weeks before the surgery [14]. 

### 1.3. Aim

The purpose of the study was:

- Assessing a relation between the degree of addiction and the level of motivation for ceasing smoking;
- Pointing out factors that have a significant impact on the motivation for ceasing smoking.

## 2. Materials and Methods

### 2.1. Design

It was a cross-sectional survey study. 

### 2.2. Study Procedures

The research was carried out at the Department of Cardiac Surgery and Vascular Surgery of the University Clinical Centre in Gdańsk in 2021. The target group consisted of 101 patients aged 33 to 81 with abdominal aortic aneurysm (AAA), peripheral arterial disease (PAD), carotid artery disease (CAD). 

In the prospective research the target group was chosen in accordance with the following criteria:

- A confirmed AAA, PAD or CAD;
- Qualification for a vascular surgery;
- An active smoker;
- Included informed consent form.

**Figure 1:**
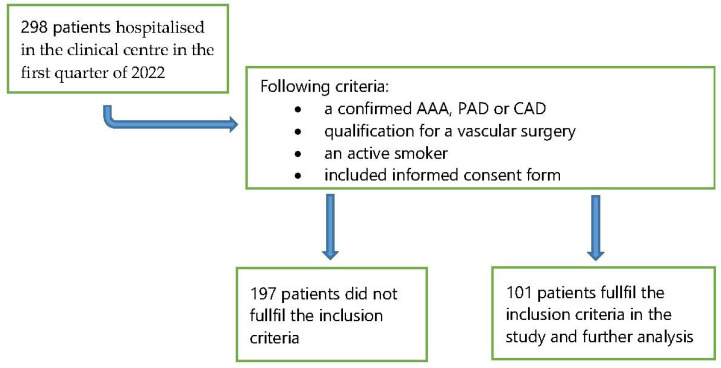
Study flow diagram showing the enrolment in the study.

The data concerning the popularity of smoking were collected in the first quarter of 2022. In that period, there were 298 patients hospitalised in the clinical centre. The analysis included active smokers (*n* = 101, 33.9%)—Figure 1.

All patients received information that in our hospital there is a total ban on smoking, advice encouraging abstinence from smoking, and then patients received nicotine plasters.

### 2.3. Preparation of the Questionnaire 

The diagnostic survey method based on the Fagerström test and Nina Schneider’s test and the original questionnaire were used.

#### 2.3.1. Fagerström Test for Nicotine Dependence (FTND)

A 6-question version of the FTND was applied. The value of score assigned to responses is from 0 to 3 points or from 0 to 1 point. The questions in the test were:

- How soon after you wake up do you smoke your first cigarette?
- Do you find it difficult to refrain from smoking in places where it is forbidden?
- Which cigarette would you hate most to give up?
- How many cigarettes per day do you smoke?
- Do you smoke more frequently during the first hours after waking than during the rest of the day?
- Do you smoke when you are so ill that you are in bed most of the day?

In the Fagerström test, the author assigned the greatest weight to the question concerning the amount of time between waking up in the morning and smoking the first cigarette, as well as the number of cigarettes smoked a day. To assess the degree of addiction, the score of the responses must be summed up. The greater the score in the FTND test, the more intensive nicotine dependence of the patient. Classification of dependence: 0–3 low, 4–6 medium, 7–10 high [15,16,17]. 

#### 2.3.2. Test of Motivation for Ceasing Smoking by Nina Schneider

The test measuring motivation for ceasing smoking prepared by the University in Los Angeles and used to test the patient’s readiness to cease smoking. The more positive responses, the greatest readiness to cease smoking. In the questionnaire, each response “yes” or “no” is scored individually. “No” means 0 points and “yes” means 1 point. To obtain the result, all scores must be summed up. The maximum possible score is 12. The motivation is considered low if the test result is below 7 points and high if the score equals or is greater than 7 points [18,19].

#### 2.3.3. Original Questionnaire

The questionnaire let us collect social and demographic data, such as sex, age, education level, place of residence, marital status, professional activeness. Further questions included the history of smoking: the initiation of nicotine addiction, the time of smoking, the number of cigarettes, attempts of ceasing smoking.

The clinical diagnosis, the type of a planned vascular surgery, and co-existing diseases were defined on the basis of the patient’s medical history.

### 2.4. Statistical Analysis

All statistical calculations were carried out using the IBM SPSS 23 statistical package and an Excel 2016 spreadsheet. Qualitative variables were presented as numbers and percentages, while quantitative variables were characterized using mean and standard deviation or median, upper and lower quartile. All variables were tested for normal distribution by the Shapiro-Wilk test. The significance of any differences between more than two groups was verified using the Kruskal–Wallis non-parametric significance test and the significance of differences between two groups, by using the Mann–Whitney. Spearman correlation test was used to verify the existence and power of the relationship between the variables. In all calculations, *p* < 0.05 was assumed as the level of significance.

### 2.5. Ethical Considerations

While collecting the data, the ethical principles set out in the Helsinki Declaration were taken into account. The research was approved by the Independent Bioethical Committee at the Medical University of Gdańsk, number NKBBN/124/2020.

## 3. Results

### 3.1. Social, Demographic, and Clinical Characteristics of the Respondents

The respondents were from 33 to 81 years of age, M = 64. Men made up the largest group (69.3%, *n* = 70). A secondary education level was declared by 51.5% (*n* = 52) of the respondents. Vocational education was reported by 24.8% of the respondents (*n* = 25). The smallest group of the respondents reported a tertiary education level—23.8% (*n* = 24). The respondents were mostly residents of towns. A total of 29.7% (*n* = 30) of the respondents lived in villages. Slightly over a half of the respondents were retired (59.4%, *n* = 60) and married (63.4%, *n* = 64). With regard to coexisting diseases, the patients usually mentioned hypertension (59.4%, *n* = 60), diabetes (34.6%, *n* = 35), and most rarely COPD (9.9%, *n* = 10) and atrial fibrillation (7.9%, *n* = 8)—Table 1 and Table 2. 

### 3.2. Smoking History 

All of the respondents were active smokers (100%, *n* = 101). They usually started smoking between 13 and 19 years of age (55.4%, *n* = 56), and rarely started younger than 13 (7.9%, *n* = 8). An average number of cigarettes smoked a day was 21. The number of years of smoking was from 15 to 60. An average number of years of smoking was 42—Table 3. Over a half of the patients (74.3%, *n* = 75) did not have any knowledge of places where they could get aid in ceasing smoking. Smoking in the past attempted to cease 61.4% (*n* = 62). The patients that used pharmacological cigarette substitutes while ceasing smoking usually used “nicotine plasters” (10.5%, *n* = 13) and “nicotine chewing gums” (8.1%, *n* = 10). The smallest number of the patients used “nicotine aerosols” (2.4%, *n* = 3). The greatest number of the patients declared that they would cease smoking within one week of the end of their hospitalisation (23.8%, *n* = 24). The smallest number declared that they would cease smoking within one year (9.9%, *n* = 10). The patients declared that their family and other closest persons did not have any impact on their decision concerning the number of cigarettes they smoke (49.5%, *n* = 50). 

### 3.3. Results of the Fagerström Test for Nicotine Dependence (FTND)

Most of the respondents showed a high level of nicotine dependence (46.5%, *n* = 47). In the case of 40.6% (*n* = 41) of the patients, nicotine dependence was moderate. The smallest number of the patients had low nicotine dependence (12.9%, *n* = 13)—Table 4. An average nicotine dependence ratio based on the Fagerström test was 6.23 ± 2.39.

### 3.4. Results of the Test of Motivation for Ceasing Smoking by Nina Schneider 

Need to increase the motivation to quit smoking 65.3% of patients (*n* = 66). Only 34.7% of the respondents (*n* = 35) were adequately motivated to cease smoking—Table 5. An average motivation for ceasing smoking ratio was 4.88 ± 2.76.

### 3.5. Social and Demographic Data versus the Level of Nicotine Dependence and Motivation for Ceasing Smoking

The statistical analysis (Spearman correlation test, Mann–Whitney U test, Kruskal–Wallis test) indicated that social and demographic variables, such as age, sex, education level, and the professional situation of the respondents, did not have a significant impact on the motivation for ceasing smoking (*p* > 0.05)—Table 6, Table 7 and Table 8. 

The statistical analysis (Spearman correlation test, Mann–Whitney U test, Kruskal–Wallis test) indicated that social and demographic variables, such as age, sex, education level, and professional situation of the respondents, did not have a significant impact on the level of nicotine dependence (*p* > 0.05)—Table 9, Table 10 and Table 11. 

### 3.6. The Number of Cigarettes Smoked a Day and the Number of Years of Smoking versus Motivation for Ceasing Smoking

**Figure 2:**
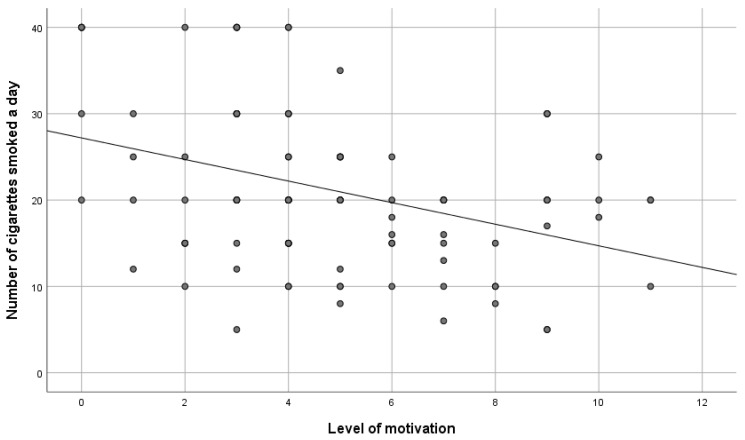
The level of motivation versus the number of cigarettes smoked a day.

The survey did not confirm a statistically significant correlation between the number of years of smoking and the level of motivation for ceasing smoking (rHO = −0.11; *p* > 0.05 (Spearman correlation tests). In turn, a negative statistically significant correlation between the level of motivation and the number of cigarettes smoked a day was identified (rHO = −0.33; *p* = 0.001). It can be interpreted that the greater the number of cigarettes smoked a day, the lower the level of motivation for ceasing smoking among the respondents—Figure 2. 

### 3.7. The Number of Cigarettes Smoked a Day and the Number of Years of Smoking versus the Level of Nicotine Dependence

**Figure 3:**
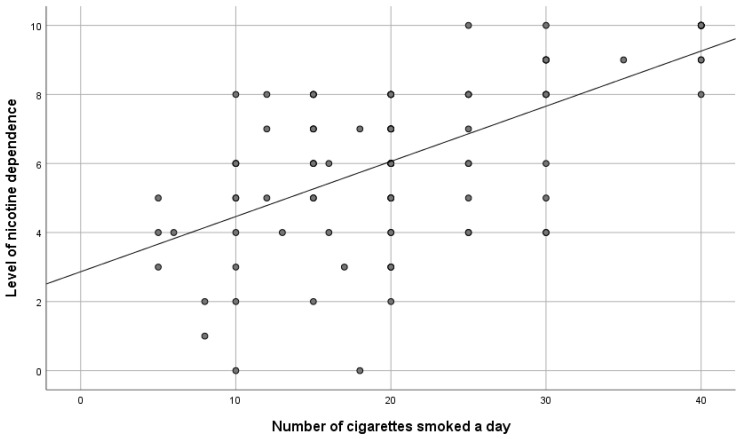
The level of nicotine dependence versus the number of cigarettes smoked a day.

To verify the hypothesis, the Spearman correlation test was made. It confirmed a positive statistically significant and moderate correlation between the level of dependence and the number of cigarettes smoked a day (rHO = 0.58; *p* < 0.001). It can be interpreted that the greater the nicotine dependence, the greater the number of cigarettes smoked a day—Figure 3. In turn, the survey did not confirm a statistically significant correlation between the number of years of smoking and the degree of nicotine dependence (rHO = 0.16; *p* > 0.05). 

### 3.8. The Number of Years of Smoking versus the Existence of Coexisting Diseases

A number of Mann–Whitney U tests were performed. The analysis indicated that the longer the smoking history, the more frequent the occurrence of chronic obstructive pulmonary disease (COPD) (Z = 2.20; *p* < 0.027). No statistically significant differences were identified between other coexisting diseases and the number of years of smoking (*p* > 0.05)—Table 12.

### 3.9. The Level of Nicotine Dependence versus Motivation for Ceasing Smoking

To verify the variable, Spearman correlation tests were performed. They confirmed a negative statistically significant correlation between the level of dependence and the level of motivation (rHO = −0.72; *p* < 0.001). It can be interpreted that the greater the nicotine dependence, the lower the motivation for ceasing smoking among the respondents.

## 4. Discussion

In Poland, there are limited data concerning smoking by patients with peripheral arterial disease (PAD), abdominal aortic aneurysms (AAA), and carotid artery disease (CAD), which would involve the level of nicotine dependence and motivation for ceasing smoking. Therefore, the authors designed a pilot survey, which allows for the assessment of that problem. The researchers proved that smoking is strictly correlated with the development of vascular diseases, including peripheral arterial disease (PAD), abdominal aortic aneurysms (AAA), and carotid artery disease (CAD) [5,6,20]. In addition, smoking contributes to the progression of vascular diseases [19]. Based on the existing guidelines, all patients to undergo vascular surgeries are recommended to cease smoking [20]. The results of the survey confirm a 40% relative reduction in the risk of all complications if the patient ceases smoking before the surgery [21].

The analysis of the authors’ survey confirmed that 33.9% of the patients were active smokers before the surgery. Over 70% of them declared that they were willing to cease smoking after the vascular surgery, usually within one week of the end of their hospitalisation. The survey performed by Assadian et al. also confirmed that a significant percentage of patients to undergo vascular surgeries are smokers. In addition, half of them still smoked in the hospital, where smoking is forbidden by law [22]. The survey performed by Sztuczka et al. indicated that in the group of PAD patients who were originally qualified for the survey, 91.4% of the respondents declared themselves to be addicted smokers. The results of the survey confirmed that the level of motivation for ceasing smoking was not dependent on the clinical progress of the disease. This means that the smokers who were to undergo a bypass surgery, due to the 3rd or 4th degree ischaemia of lower limbs, are less inclined to look for professional help and cease smoking [18]. The survey conducted in the Canadian centre confirmed that 33.5% of the respondents were smokers during the perioperative period. After the yearly observation of the patients who smoked before the surgery, 41.6% ceased smoking and 58.4% did not do that [23].

The analysis of the survey hereunder did not confirm the impact of social and demographic factors, such as sex, age, education level, on the level of nicotine dependence and motivation for ceasing smoking. The survey conducted by the Canadian centre confirmed that male patients smoked before the surgery more frequently. The sex was also statistically related with the higher ratios of ceasing smoking among women [23]. Ponczek et al. confirmed the impact of social and demographic factors on the level of motivation for ceasing smoking. The authors of that survey conclude that the higher education level, the greater readiness to cease smoking. In addition, that survey confirmed that the degree of nicotine dependence among the respondents with vocational education was greater [24]. 

The survey conducted by Smeds et al. indicated that 22% of the patients were active smokers before the vascular surgery. The smokers that smoked less than 10 cigarettes a day were less nicotine dependent (*p* = 0.0001) and more inclined to consider ceasing smoking [25]. The survey hereunder also confirmed that the greater the number of cigarettes smoked a day, the higher the level of nicotine dependence and the lower the motivation for ceasing smoking. In the survey conducted by Walewska et al., it was also confirmed that nicotine dependence increases together with the growth of stress, as a result of which the patient smokes a cigarette and the level of motivation for ceasing smoking decreases [26].

The surveys prove that nicotine is the major—however, not the only—reason for the development of chronic obstructive pulmonary disease (COPD) [27,28,29,30] and there seems to be a genetic predisposition, which increases the risk in the case of certain patients [31]. COPD develops in the case of 10–20% of smokers [32,33,34]. In our surveys, the respondents with a greater number of years of smoking were confirmed to be diagnosed with COPD much more often.

To obtain better results, a special attention should be paid to motivating the patient adequately to attempt to cease smoking and refrain from smoking as long as possible. It must be noted that motivation is a dynamic process and can be strengthened or maintained potentially in the case of each patient [22,35]. Our own data indicate that a significant number of patients keep on smoking despite their diagnosis. As ceasing smoking is desirable, all patients should be encouraged to do it [2,36]. 

## 5. Limitations

Our study has several limitations. The overall sample size was small, so the results of the study cannot be generalized. Additionally, the level of addiction and the motivation to quit smoking may be influenced by other factors that we did not take into account. We believe that the next test should take into account factors, such as financial considerations, free time, stress, and having loved ones who smoke.

In another test, the competence of vascular surgeons and vascular nurses in the area of counseling and smoking cessation treatment should be assessed. This will enable the development of training programs for doctors and nurses in the effective implementation of the diagnostic protocol and the treatment of nicotine addiction.

Despite the limitations, the results of these studies show the magnitude of the problem of nicotine addiction in patients with vascular diseases and can be used to design and implement smoking cessation programs.

## 6. Conclusions

Most patients undergoing vascular surgeries showed a high or moderate degree of nicotine dependence and low motivation to quit smoking. The greater the addiction to nicotine, the lower the motivation to quit smoking. Social and demographic factors do not affect the degree of nicotine addiction and the motivation to quit smoking. Years of smoking had an impact on the incidence of chronic obstructive pulmonary disease (COPD).

## 7. Implications for Practice

Based on the surveys and information coming from other researchers, special attention should be paid to the need of implementing comprehensive intervention and prevention measures in the case of patients qualified for vascular surgeries in order to enable the addicted smokers to cease smoking as fast as possible. The smoking cessation programme combined with intensive consultancy and a nicotine substitute therapy should be offered to all patients during the period they are prepared to a surgery. The traditional approaches focused on the postoperative period to rehabilitate and change the patient’s lifestyle. However, the latest evidence proves that the preoperative period is the best time to intervene. Hospitalised patients should have their smoking status identified and recorded systematically and obtain specific help in order to attempt to cease smoking. Smoking in the past and at present should be measured by the use of questionnaires. The purpose of such an approach is to enable the patients to cease smoking before the surgery.

## Figures and Tables

**Table 1:** Socio-demographic characteristics of the studied group.

Characteristics of the Respondents	N	%
**Sex:**		
Woman	31	30.7
Men	70	69.3
**Education:**		
Vocational	25	24.8
Secondary	52	51.5
University degree	24	23.8
**Employment status:**		
Retired	60	59.4
Sickness pension	22	21.8
Employed	14	13.9
Unemployed	5	5
**Place of residence:**		
Village	30	29.7
City	71	70.3
**Marital status:**		
Not married	9	8.9
Married	64	63.4
Widow/widower	17	16.8
Divorced	11	10.9

N—number of respondents.

**Table 2:** Clinical characteristics of the studied group.

Characteristics of the Respondents	N	%
**Clinical diagnosis**		
Peripheral arterial disease	66	65.3
Abdominal aortic aneurysm	23	22.8
Carotid artery disease	12	11.9
**Coexisting diseases**		
Diabetes	35	18.8
Arterial hypertension	60	32.3
Chronic obstructive pulmonary disease	10	5.4
Cardiac failure	11	5.9

N—number of respondents.

**Table 3:** Smoking history.

Results	N	Min	Max	M	SD
Number of cigarettes smoked a day	101	5	40	21.09	9.34
Number of years of smoking	101	15	60	42.08	10.79

N—number of respondents M—mean value; SD—standard deviation.

**Table 4:** FTND results.

Results	N	%
Low dependence	13	12.9
Moderate dependence	41	40.6
High dependence	47	46.5

N—number of respondents.

**Table 5:** Results of the test of motivation for ceasing smoking by Nina Schneider.

Results	N	%
Need to improve motivation	66	65.3
High motivation	35	34.7

N—number of respondents.

**Table 6:** Motivation for ceasing smoking vs age.

Motivation for Ceasing Smoking	N	rHO	*p*
Age	101	−0.06	0.524

N—number of respondents; *p*—level of significance; rHO—Spearman’s rank correlation.

**Table 7:** Motivation for ceasing smoking vs sex.

Motivation for Ceasing Smoking vs. Sex	N	Me	Q_25_	Q_75_	Rang	Z	*p*
Woman	31	5	3	7	54.35	0.77	0.44
Men	70	4	3	7	49.51		

N—number of respondents; Q_25_; Q_75_ = upper and lower quartile; Me—median; Rang—range; Z—normal distribution, the result of the Z test; *p*—level of significance.

**Table 8:** Motivation for ceasing smoking vs education level and employment status.

**Motivation for Ceasing Smoking vs. Education Level**	**N**	**Me**	**Q_25_**	**Q_75_**	**Rang**	**H**	**df**	***p***
Vocational	25	4	3	7	50.52	0.48	2	0.784
Secondary	52	4.5	3	7	52.72			
University degree	24	4	3	7.25	47.77			
**Motivation for ceasing smoking vs. employment status**	**N**	**Me**	**Q_25_**	**Q_75_**	**Rang**	**H**	**df**	***p***
Retired	60	4	3	6	47.88	3.97	3	0.264
Sickness pension	22	4	3	8	49.2			
Employed	14	6	3.75	9	62.75			
Unemployed	5	6	4	7.5	63.4			

N—number of respondents; Q_25_; Q_75_ = upper and lower quartile; Me—median; Rang—range; *p*—level of significance; df—degrees of freedom.

**Table 9:** The level of nicotine dependence vs age.

The Level of Nicotine Dependence	N	rHO	*p*
Age	101	0.05	0.958

N—number of respondents; *p*—level of significance; rHO - Spearman’s rank correlation.

**Table 10:** The level of nicotine dependence vs sex.

The Level of Nicotine Dependence vs. Sex	N	Me	Q_25_	Q_75_	Rang	Z	*p*
Woman	31	6	4	8	47.13	0.89	0.373
Men	70	6	5	8	52.71		

N—number of respondents; Q_25_; Q_75_ = upper and lower quartile; Me—median; Rang—range; Z—normal distribution, the result of the Z test; *p*—level of significance.

**Table 11:** The level of nicotine dependence vs. education level and employment status.

**The Level of Nicotine Dependence vs. Education Level**	**N**	**Me**	**Q_25_**	**Q_75_**	**Rang**	**H**	**df**	***p***
Vocational	25	6	5	9	52.82	0.22	2	0.895
Secondary	52	6.5	5	8	49.72			
University degree	24	6	4	8.75	51.88			
**The level of nicotine dependence vs. employment status**	**N**	**Me**	**Q_25_**	**Q_75_**	**Rang**	**H**	**df**	***p***
Retired	60	6	4.25	8	51.53	0.39	3	0.942
Sickness pension	22	6	4.25	8.25	49.66			
Employed	14	7	4.75	8	53.14			
Unemployed	5	6	4	7.5	44.5			

N—number of respondents; Q_25_; Q_75_ = upper and lower quartile; Me—median; Rang—range; *p*—level of significance; df—degrees of freedom.

**Table 12:** Years of smoking and coexisting diseases.

**Number of Years of Smoking Versus Diabetes**	**N**	**Me**	**Q_25_**	**Q_75_**	**Rang**	**Z**	***p***
No	66	42.5	34.75	50.5	51.64	0.3	0.761
Yes	35	43	36	49	49.79		
**Number of years of smoking versus arterial hypertension**	**N**	**Me**	**Q_25_**	**Q_75_**	**Rang**	**Z**	***p***
No	41	41	31.5	49.5	47.35	1.03	0.301
Yes	60	43.5	36	50	53.49		
**Number of years of smoking versus chronic obstructive ** **pulmonary disease**	**N**	**Me**	**Q_25_**	**Q_75_**	**Rang**	**Z**	***p***
No	91	42	34	50	48.87	2.2	**0.027**
Yes	10	49.5	44	54	70.4		
**Number of years of smoking versus cardiac failure**	**N**	**Me**	**Q_25_**	**Q_75_**	**Rang**	**Z**	***p***
No	90	43	35	50	52.15	1.13	0.259
Yes	11	40	30	50	41.59		

N—number of respondents; Q_25_; Q_75_ = upper and lower quartile; Me—median; Rang—range; Z—normal distribution, the result of the Z test; *p*—level of significance.

## Data Availability

A dataset will be made available upon request to the corresponding authors one year after the publication of this study. The request must include a statistical analysis plan.
